# Neuromodulatory systems

**DOI:** 10.3389/fncir.2013.00036

**Published:** 2013-03-26

**Authors:** Gerhard Werner, Bernhard J. Mitterauer

**Affiliations:** ^1^Department of Biomedical Engineering, University of TexasAustin, TX, USA; ^2^Forensic Psychiatry and Guenther Archives, University of SalzburgWals, Austria

**Keywords:** neuroglia, extracellular fluid, neuromodulation, multifractals, neuromodulatory systems

## Abstract

We examine the interactions and interdependencies between Neuroglia, the Brain-Cell Microenvironment, and the processes commonly subsumed under Neuromodulation. The interactions of the component processes covering a wide spectrum of frequencies are designated as Neuromodulatory Systems (NMS). This implies NMS's scale-invariance as the capacity of linking actions across many time scales, and self-similarity at any scale. These features endow NMS with the ability to respond adaptively to neural impulse traffic of an unpredictably wide frequency spectrum. In this preliminary perspective, the components of NMS are only outlined based on concepts of Complex Systems Dynamics. However, their interactions must be formally elaborated in further investigations.

## Introduction

We address three aspects of neuroscience, each for long being largely overshadowed by the Neuron Doctrine's hegemony (Bullock et al., 2005): Neuroglia (including here also the systems of neuronal and glial Gap Junctions), Extracellular Fluid in neural tissue (“Brain-Cell Microenvironment”: Nicholson, 1980), and neuromodulatory processes. Our leading notion is that the functional state of neurons, individually and in assemblies, is determined by a set of variables (ion conductances and membrane currents, thresholds for neural discharges, synaptic potentials, ion channel kinetics, etc.), whose values at any one time are to varying degrees affected by interactions and interdependencies of these three components, locally as well as globally, and at largely different time scales. In the section “Background,” we review essential aspects of each of these components separately. This is to provide the basis for our principal objective to analyze in the “Discussion” section the global dynamics of the complex system these components jointly compose, covering a wide range of temporal scales that is characteristic of Multifractals. Accordingly, self-similarity and the absence of any specific time scale ensure instant and automatic adaptation to neural impulse traffic over a wide range of frequencies.

## Background

### Gap junctions and neuroglia

Diffusive coupling by Gap Junctions between various interneuron types and neuroglia from cells in neocortex is now firmly established (Simon et al., 2005), as is their virtually boundless distribution (Fukuda, 2007). Simulation studies determined their role for supporting synchronous oscillations (Traub et al., 1999; Lewis and Rinzel, 2000), and identified complementary interrelations with chemical synapses in interneuronal networks (Kopell and Ermentrout, 2004). Electrical coupling between axons is also amply documented (Debanne and Rama, 2011), providing the opportunity for fast and efficient transfer of action potentials for generating highly coherent output pathways of neuronal networks.

Neurochemists generated an avalanche of data, promoting Astrocytes (Verkhratsky and Butt, 2007), one of the members of the macro-glia family, to full partnership with pre- and postsynaptic neurons in the “Tripartite Synapse” (Araque et al., 1999). This has become a fertile concept for characterizing the complex and reciprocal patterns of interactions between astrocytes and neurons, reviewed by Araque and Navarrete (2010) and Halassa and Haydon (2010). The dynamics of these interactions is sustained by the astrocytes expressing receptors for virtually all important neurotransmitters (Kettenmann and Steinhauser, 2005), providing multiple opportunities for engaging neuron-astrocyte complexes at many target points (Fields and Stevens-Graham, 2002; Auld and Robitaille, 2003; Perea et al., 2009). A prevalent feature of astrocytes' role is their participation in the dynamics of calcium in extracellular space on two different space and time scales. For local short-term interaction, astrocytes (although not electrically excitable) respond to glutamate liberated at presynaptic junctions with calcium spikes which, in turn, release additional glutamate and ATP to neighboring neurons (Smith and Pereda, 2003) for integrating coincident activity from different dendrites in the same tissue volume (Parpura and Haydon, 2000; Bezzi et al., 2001). Furthermore, activity related changes of calcium levels within astrocytes contribute to mobilizing various transmitters and transmission-related substances (Perea and Araque, 2010). Globally and on longer time scales, intercellular propagation of Calcium waves (Cornell-Bell et al., 1990; Charles, 1998; Harris and Timofeeva, 2010) can support long-range signaling (Giaume and Venance, 1998; Kuga et al., 2011). Recent evidence from the family of connexins suggest that the astrocyte system constitutes a network of communicating cells with definite spatial organization (Pereira and Furlan, 2010) where intercellular communication is controlled by endogenous signals (Giaume and Liu, 2011).

The dynamics of neuron glia interaction is complicated by two circumstances: one, due to activity-dependent morphological changes of astroglia processes ending at synaptic regions (Hirrlinger et al., 2004; Theodosis et al., 2008; Fellin, 2009); and, the second, due to a complex anatomical organization of spatial non-overlapping domains with limited interdigitation of processes from adjacent cells (Bushong et al., 2002; Ogata and Kosaka, 2002; Halassa et al., 2007). Each domain encompasses some 2 million synapses in human brain (Oberheim et al., 2008) as an area of the neuropil that is controlled by a single astrocyte. Moreover, parts of this territory can be controlled autonomously by specialized astrocyte microdomains of filipodia with distinct motility (Volterra and Meldolesi, 2005). Groups of neurons are also enwrapped by a layer of lattice-like material: this Perineuronal Net forms stable complexes surrounding synapses (Faissner et al., 2010; Kwok et al., 2011), seemingly affecting short-term synaptic plasticity (Frischknecht et al., 2009). On a modular basis, computational simulations of different manifestations of astrocyte-neuron interactions contribute to gaining some insight into their functions (Nadkarni et al., 2008; Goldberg et al., 2010; De Pitta et al., 2011). On the basis of theoretical arguments, Mitterauer (2012) attributed a structural organization in the form of logical functions to the tripartite synapse and astrocyte domain organization, suggesting its role in the economy of normal and pathological brain functions (Mitterauer, 2010; Mitterauer and Kofler-Westergren, 2011).

### Brain-cell microenvironment (extracellular fluid)

Extracellular fluid's coming-of-age is closely associated with the work of Kuffler and Nicholls (1966) that identified the diffusion of ions and various neuroactive substances in intercellular clefts of neural tissue. This theme was again taken up by Vizi and Labos (1991), documenting non-synaptic interaction in nervous tissue, subsequently discussed in detail by Agnati et al. (1995) and Zoli and Agnati (1996), suggesting to view intercellular communication among cells in the nervous system in two complementary reference frames, one as “wiring” transmission, the other as “volume” transmission: the former being transmission of excitation between synaptically connected neurons, the latter attributing diffusive distribution of various ions, neuropeptides, and neurotransmitters to extracellular fluid surrounding neurons. A wealth of experimental data, notably with the effects of ion accumulation in the extracellular fluid following tetanic nerve activity corroborated this conjecture (Frankenhaeuser and Hodgkin, 1956; Egelman and Montague, 1998). In a computational model of a Reaction-Diffusion system, Werner (2005) demonstrated that tetanic stimulation of a group of neurons causes extracellular accumulation of Calcium ions which induces spreading activity patterns in surrounding unstimulated neurons. More recently, Froehlich et al. (2006) showed that diffusive modulation of extracellular potassium concentration induces state transitions in neurons with distinct changes in oscillatory patterns. Changes of diffusive coupling in neural networks can change normal, and precipitate pathological, activity patterns (Ullah et al., 2009; Durand et al., 2010). The relevance of non-synaptic diffusion neurotransmission was further extended and refined by Bach-Y-Rita (1995). However, diffusion of neuroactive substances is slowed down by geometric tortuosity and viscosity of macromolecules in extracellular space (Rusakov and Kullmann, 1998; Hrabe et al., 2004). By changing their geometric shape, dendritic spines can dynamically regulate diffusion in their vicinity (Biess et al., 2007).

### Neuromodulatory processes

The notion of Neuromodulation originated with a diversity of observations that could not be accounted for within the established principles of synaptic transmission with transmitter substances exclusively acting locally at synaptic sites (Kaczmarek and Levitan, 1987; Katz, 1999). For present purposes, we suggest reserving the term “Neuromodulation” to designate the composite system of all processes affecting synaptic transmission, in distinction from individual neuromodulatory processes in the narrower sense, as listed in the following. The foundational insights on operational principles of neuromodulation originated with investigating the polyvalence of neural network functions in crustaceans. A succession of comprehensive reviews by Getting (1989), Harris-Warrick and Marder (1991), and Marder and Calabrese (1996), summarize the repertoire of neuronal functions attributable to the modulating action of extrasynaptic processes on synaptic transmission. They include effects on synaptic efficacy and presynaptic transmitter release, intrinsic neuronal properties, changes of network connectivity, coupling of neural oscillators, and filtering sensory input, and spike-time dependent plasticity (Pawlak et al., 2010). All known neurotransmitter substances are involved in these effects, as are a multitude of peptides (Nusbaum, 2002; Nassel, 2009).

Reports of neuromodulatory effects in higher functions of vertebrates followed the crustacean work in rapid sequence: Hasselmo and associates produced evidence for forebrain cholinergic neuromodulation of Cognition (for review see: Hasselmo and Sarter, 2011). Ascending brain stem neuromodulatory systems (NMS) of vertebrate brains became implicated in learning mechanisms (Doya, 2002), in adaptive behavior (Krichmar, 2008), and in emotional control processes (for a recent example, see Cools et al., 2007). Central pattern generators are subject to neuron modulation in vertebrates as they are in invertebrates (Dickinson, 2006). In its totality, the accumulated observational evidence mandates expanding the classical view of a relatively static neuronal “wiring diagram” to a dynamic system subject to ongoing tuning and reconfiguring by a biochemical network of modulators, effective over a wide range of temporal, and spatial scales (Brezina, 2010). Combining experimental observations with computational simulations reveals the combinatorial richness of the modulatory network for generating functionally appropriate and adaptive behavior (Brezina et al., 2000; Proekt et al., 2004; Stern et al., 2007).

The multitude of phenomena described as neuromodulation fall into two fundamentally different categories (Marder and Thirumalai, 2002): intrinsic neuromodulation is the condition of the modulator being released by some of the same neurons that are also part of the circuit they modulate (Katz and Frost, 1996). Hansson and Rönnbäck (1994) review several instances of intrinsic modulation of synaptic transmission by astrocytes, related to release and uptake of glutamine at synaptic sites. Events at the “Tripartite Synapse” fall also in this category. Extrinsic modulation, on the other hand, consists in activity of functionally distinct system processes outside of and parallel to the actual synaptic activity, relying on the storage and transport of neuroactive substances in the extracellular fluid compartment. Most of the effects of neuroglia must be attributed to this category. Beyond regulating merely one synaptic region, extrinsic modulation can globally organize ensembles of circuits, and usually works at a time course up to several seconds rather than the msec's of synaptic actions of intrinsic modulation.

## Discussion and conclusion

In the following discussion, we will refer to the totality of the interacting complex of glia, extracellular fluid and the processes of neuromodulation as NMS. For formulating ideas about NMS, it must of course not be overlooked that neurons themselves (individually and as assemblies) are integral participants, active by contributing to the flux of neuroactive substances in extracellular space, and passive by being affected by them. Since our aim is to characterize the function of NMS at a global level, we take a coarse-grained, non-reductive perspective. This sets our approach apart from studies of stochastic synaptic processes at the molecular level (Ribrault et al., 2011) and the multiscale analysis of molecular processes at cellular levels (for a recent overview: see Holcman, 2012).

We make the biologically plausible assumption that each process in the chain of neuromodulatory events can be considered a chemical rate process with exponential decay. Relaxation rates vary over at least a thousand-fold range: from milliseconds at the liberation of transmitter substances at intrinsic modulation, to many seconds of chemically mediated astrocyte network reconfigurations and propagating calcium waves, with the numerous extrinsic modulatory processes exhibiting intermediate rates. This situation invites applying the observation of Hausdorff and Peng (1996) that systems presenting time series with widely differing scales of component regulatory mechanisms summate to a system's power-law (1/f) scaling, suggestive of its fractal character. Although there is no definitive mathematical proof presently available that time-scale free functions emerge from superposition of independent relaxation processes, there exists a range of physical mechanisms that do in fact show such micro- to macroscopic conversion, generally in the context of fractal time series (Marom, 2010). Moreover, numerical analyses of Montroll and Shlesinger (1982) established that macroscopic scale-free functions emerge, provided the independent microscopic relaxation processes are of sufficiently large variance, as they are in NMS. This principle was subsequently applied by Anderson (2001) to ascertain the power-law dependency as an emergent property of systems that contain several exponentially decaying traces and was further extended by Fusi et al. (2005) and Drew and Abbott (2006) to include cascading exponential processes, the latter for sensory adaptation. However, NMS contain too many unknown rate constants to attempt numerical simulation and determination of a power law exponent. Thus, we need to confine the discussion to the exposition of plausible principles and analogies.

Placing the function of NMS into the domain of fractal time series allows gaining significant insights into the dynamic properties. In the first place, the scale-invariance is identified as the property of relating the behavioral elements of NMS in time across multiple time scales. This is a characteristic empirical feature of a large number of complex physiological phenomena (West, 2010). It implies the global system's capacity for linking actions across many time different scales of the constituent processes: there is no privileged time scale, and the system's temporal performance is self-similar at any scale. This property endows the system with the ability to respond adaptively to perturbations (external events) over a wide range of their temporal patterns, and enables adaptation to impinging neural impulse trains that vary unpredictably over a wide range of time scales (Werner, 2010, 2011). However, if the special formal properties of self-similarity, etc. are not present, then what we are left with is a collection of processes on multiple time scales, which can therefore respond to perturbations on various time scales individually, but not necessarily as a coherent system.

In the application cited in the foregoing, the systems were sufficiently small that power-laws with only one exponent were considered adequate. Hence, they fall into the category of Monofractals. Granting, however, the plausibility of the suggested approach, we consider it necessary to introduce a refinement: characteristic time scales of the NMS component processes known to extend over a thousand fold range (as stated earlier) render fitting a power-law function with only one exponent unlikely. Accordingly, several power-functions with different exponents, each covering a section of the entire spectrum of scales, are required. This places NMS into the category of Multifractals (Stanley and Meakin, 1988; Mandelbrot, 1999), commonly thought indispensable for very large systems (for instance: Geophysics: Mandelbrot, 1999), but also successfully applied in numerous biological systems (West, 2010; West and Grigolini, 2010). This underscores the wide range of temporal scales to which systems with fractal characteristic can successfully adapt (Werner, 2010).

## A final thought

Given the function NMS are to satisfy the requirements stipulated in the foregoing, it is from an engineering point of view surely extremely clumsily designed, with many redundancies and duplications of functions. Why is this so? It gives the impression that NMS in the present state may represent stages, one stage superimposed on the other as if to attain a progressively higher degree of robustness and stability for assuring secure contact with an ever-changing and unpredictable environment: perhaps many stages of consecutive “tinkering”; yet, seemingly preserving modular semi-autonomy. Admittedly, in this first perspective the components of NMS are only outlined. Their interactions must be formally elaborated in further investigations.

### Conflict of interest statement

The authors declare that the research was conducted in the absence of any commercial or financial relationships that could be construed as a potential conflict of interest.
